# Overnight Fasting and Body Weight: Emulated Target Trial Using Cancer Prevention Study-3 Data

**DOI:** 10.3390/nu17091559

**Published:** 2025-04-30

**Authors:** Valeria Elahy, Ying Wang, W. Dana Flanders, Charlie Zhong, Marjorie L. McCullough

**Affiliations:** 1 Department of Population Science, American Cancer Society, Atlanta, GA 30303, USA; ying.wang@cancer.org (Y.W.);; 2 Department of Epidemiology, Rollins School of Public Health, Winship Cancer Institute, Emory University, Atlanta, GA 30322, USA

**Keywords:** fasting, meal timing, body weight, emulated target trial

## Abstract

**Background/Objectives**: Intermittent fasting has gained attention in managing weight, yet its long-term effects remain unclear. We examined the impact of overnight, before-sleep, and after-sleep fasting on body weight over two years using data from the Cancer Prevention Study-3 Diet Assessment Substudy. **Methods**: We emulated three target trials in 457 adults without diabetes or cancer. Participants were assigned to fasting strategies of <12 vs. ≥12 h overnight, <4 vs. ≥4 h before sleep, and <1 vs. ≥1 h after sleep at baseline (2016). Mean body weight 2 years post baseline was estimated using marginal structural models with stabilized inverse probability weights, adjusting for pre-baseline covariates. **Results**: After two years (median [IQR]: 2.0 [1.8–2.0] years), the estimated mean body weight was 79.4 kg (≥12 h overnight) vs. 78.9 kg (<12 h overnight) (mean difference: 0.4 kg; 95% CI: −4.1 to 4.7); 79.4 kg (≥4 h before sleep) vs. 77.5 kg (<4 h before sleep) (mean difference: 1.9 kg; 95% CI: −0.4 to 4.1); and 79.8 kg (≥1 h after sleep) vs. 78.9 kg (<1 h after sleep) (mean difference: 0.9 kg; 95% CI: −4.3 to 4.4). Among men, overnight fasting ≥ 12 h showed a higher weight (100.9 kg vs. 83.9 kg, mean difference: 17.0 kg; 95% CI: 10.8, 23.1), whereas, among women, it was estimated weight was lower (74.3 kg vs. 77.1 kg, mean difference: −2.8 kg; 95% CI: −6.8, 1.2). **Conclusions**: Overall, overnight fasting alone may not substantially influence body weight, but sex-specific differences suggest a need for further investigation.

## 1. Introduction

Intermittent fasting has gained considerable attention as a dietary strategy for managing body weight and metabolic health. Data from the 2015–2016 NHANES show that over half of Americans fast for at least 12 h overnight [1]. Despite this, obesity rates continue to rise, with one in five adults in every state living with obesity in 2023 [2,3].

While numerous randomized trials have investigated the effect of time-restricted eating (TRE), a specific form of intermittent fasting, on body weight, results remain inconsistent, highlighting several key limitations in current evidence [4,5,6]. Most trials target specific populations, such as athletes or individuals with metabolic conditions, with typically small sample sizes (16–174 participants) and limited durations (none exceeding 12 months) [5,7,8,9]. Additionally, these trials primarily focus on late TRE, leaving early TRE largely unexplored despite its potential for greater metabolic benefits [10]. This underscores the need for larger, longer-term randomized trials in diverse populations to evaluate the effects of fasting on body weight, which are not feasible [4].

To address these gaps, we emulated a target trial, leveraging data from 457 participants in the prospective Cancer Prevention Study-3 Diet Assessment Substudy (CPS-3 DAS). An emulated target trial is an analysis of observational data that can be viewed as an attempt to mimic a hypothetical pragmatic randomized trial to answer a causal question [11]. Several studies have successfully applied this framework to evaluate the effects of lifestyle exposures [12], including dietary ones [13,14], on mortality and chronic disease outcomes, demonstrating that, when properly implemented, effect estimates from an emulated target trial closely align with the results from an actual RCT [15]. In this study, we examined the effect of overnight fasting (<12 h vs. ≥12 h), before-sleep fasting (<4 h vs. ≥4 h), and after-sleep fasting (<1 h vs. ≥1 h) on body weight two years after treatment assignment. To emulate randomization and ensure exchangeability, we apply marginal structural models (MSMs) with inverse probability weighting (IPW), controlling for baseline and pre-baseline confounders [16]. By assuming positivity and consistency, we aim to estimate causal effects, closely mimicking the conditions of a randomized trial [17].

## 2. Materials and Methods

A target trial is a pragmatic, hypothetical, randomized controlled trial that we would like to conduct to answer a causal question of interest [11,18]. We designed this observational analysis as an attempt to emulate three target trials of overnight fasting strategies and body weight. Target trial emulation has two steps: (1) target trial specification (setting up of the study protocol, outcome definition, and causal contrasts; (2) emulation of the components of the target trial protocol using observational data.

### 2.1. Target Trial

The key protocol components of this target trial are outlined in Table 1 (left column). Eligibility criteria for all three trials include adults without a history of cancer or type 2 diabetes who are not following weight-loss diets at baseline. We would exclude pregnant women [19] (due to their special nutritional needs and pregnancy-related body weight and metabolic changes), night shift workers [20] (irregular sleep patterns and eating times among night shift workers can significantly impact their metabolic health, making it challenging to isolate the effects of fasting interventions), and those with irregular sleep patterns (night sleep <4 or >14 h, starting night sleep after 5:00 a.m. or before 5:00 p.m.), irregular eating patterns (≤1 or >8 meals/day or eating during night sleep), implausible energy intake (<800 or >4200 kcal/day for men and <500 or >3500 kcal/day for women), or extreme body mass index (<18.5 kg/m^2^ or >50 kg/m^2^).

Three independent target trials would assess the effect of the following fasting strategies of interest:

1. Overnight fasting trial: <12 h vs. ≥12 h;
2. Before-sleep fasting trial: <4 h vs. ≥4 h;
3. After-sleep fasting trial: <1 h vs. ≥1 h.

Each trial independently assesses the effects of overnight, before-sleep, and after-sleep fasting interventions on body weight [11].

These fasting intervention cutoff points were chosen based on several considerations. The ≥12 h overnight fasting duration aligns with common practices in intermittent fasting protocols and is often recommended for metabolic health benefits [21]. Several studies and reviews have shown that a 12-h fasting period aligns with common intermittent fasting protocols and offers potential metabolic health benefits and minimal negative outcomes [22]. The ≥4 h before-sleep fasting duration cutoff was selected to investigate the effects of avoiding food intake close to bedtime, based on studies showing that late-night eating is linked to poorer metabolic outcomes [23,24]. The ≥1 h after-sleep fasting duration was chosen to explore the potential impact of delaying the first meal of the day on weight management. These chosen cutoff points represent practical and achievable fasting durations for the general population, promoting adherence and real-world applicability. Time-restricted eating schedules like 12:12 and 16:8 are popular and considered flexible, making them suitable for various lifestyles and ensuring that fasting durations can be easily integrated into daily routines [25]. In addition, these cut-off points were the closest to the mean durations in the CPS-3 cohort eligible for this emulated trial.

Participants would be randomly assigned to longer or shorter strategies of each intervention and asked to follow them for two years. Follow-up would be from intervention assignment until death, loss to follow-up, or administrative end of follow-up two years after the treatment assignment. The primary outcome of interest would be the body weight self-reported two years after the assignment of treatment strategies, aiming to assess the intention-to-treat effects of intervention by comparing shorter to more extended fasting strategies for each intervention.

### 2.2. Target Trial Emulation Using Observational Data

We emulated the above target trial using observational prospective CPS-3 DAS data.

Between 2006 and 2013, the CPS-3 study enrolled over 300,000 adults across 36 states, Puerto Rico, and the District of Columbia to study cancer risk factors, focusing on lifestyle, genetic, and environmental influences [26]. The CPS-3 DAS, specifically designed to validate the cohort’s modified Food Frequency Questionnaire (FFQ), enrolled 866 participants and collected data on meal and sleep timing, diet, physical activity, alcohol consumption, and body weight in 2015 and 2016 [27,28]. Diet quality, energy intake, and alcohol consumption were ascertained from the FFQ data in 2015 and 2016 using the 2015 Healthy Eating Index score to assess diet quality [21]. Participants’ weight was self-reported in 2015, 2016, and 2018 [26], with self-reported measurements previously validated against in-person measurements in CPS-3 [29]. The 2015 and 2018 surveys collected demographic, behavioral, medical history, and anthropometric data [30].

**Figure A1:**
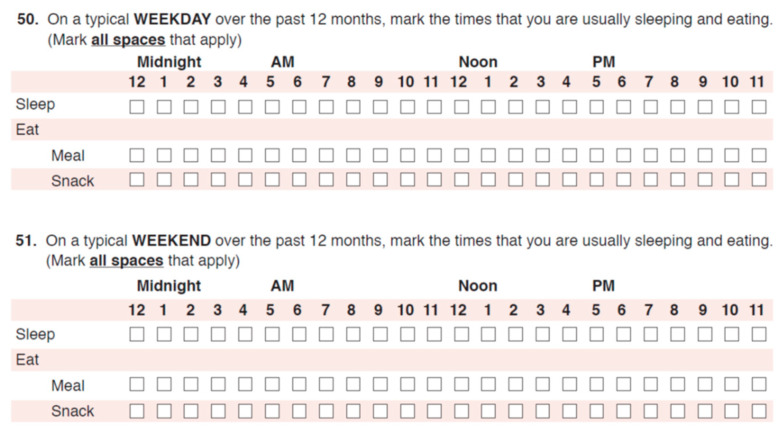
Meal and sleep timing grid. Participants marked all hours they were usually sleeping, eating meals, or snacking over a typical weekday and weekend day (midnight-11 p.m.), as used to derive fasting intervals in CPS-3 DAS.

In CPS-3 DAS, overnight, before-sleep, and after-sleep fasting durations were assessed using a validated CPS-3 24-h meal and sleep timing grid in 2015 and 2016 (Figure A1) [21]. Participants were asked: “On a typical weekday over the past 12 months, mark the times that you are usually sleeping and eating (Mark all spaces that apply)” and “On a typical weekend over the past 12 months, mark the times that you are usually sleeping and eating (Mark all spaces that apply)”. Separate rows corresponded to each hour from 12 midnight to 11 pm, and an eating occasion was defined as any instance of food intake, including meals or snacks. This grid was validated against up to six 24-h dietary recalls over a one-year period. As measured by sensitivity and specificity, the area under the receiver operating characteristic curve (AUC) for overnight fasting ≥12 h was 0.744 for weekdays and 0.718 for weekends; for ≥14 h fasting, AUCs were 0.776 and 0.640, respectively. Spearman correlations per hour of overnight fasting were 0.51 (95% CI: 0.45–0.56) for weekdays and 0.39 (95% CI: 0.31–0.46) for weekends [21].

Overnight fasting was defined as the longest interval of no eating surrounding the longest sleep period. For each grid (weekday and weekend), we calculated the time from the end of the last marked eating occasion before the onset of the longest sleeping period to the start of the first marked eating occasion after that sleep [31]. We used the end of the hour box for the last eating occasion and the start of the hour box for the first eating occasion when computing this interval. Before- and after-sleep fasting were each calculated per grid and then averaged over the week. For each grid (weekday and weekend), before-sleep fasting was defined as the interval from the end of the last eating occasion (end of that hour block) to the beginning of the longest sleep period, and after-sleep fasting as the interval from the end of the longest sleep period to the start of the first eating occasion the next day (start of that hour block). We computed a weighted 7-day average fasting duration as (5 × weekday + 2 × weekend)/7 and used this value to assign participants to <12 h or ≥12 h of overnight fasting, <4 h or ≥4 h of before-sleep fasting, and <1 h or ≥1 h for after-sleep fasting.

### 2.3. Modifications to the Target Trial Protocol

Several modifications had to be applied to the target trials to align these emulated trials with the CPS-3 DAS data (Table 1, right column).

Because meal and sleep timings were only assessed in 2015 and 2016, inquiring about the participants’ habits in the past 12 months before the assessments and not about their intended fasting durations, we assumed that the meal and sleep timing grid accurately represented both the average and the intended fasting durations for the following year. We used 2016 as the baseline, with adjustments made for fasting durations reported in 2015 as pre-baseline fasting exposure to emulate randomized treatment assignments.

We identified individuals who met all eligibility criteria for the target trial and excluded participants with missing baseline and pre-baseline fasting duration or other covariates. Eligible individuals were followed from their DAS 2016 questionnaire return to their CPS-3 2018 questionnaire return with a mean follow-up duration of 704.7 ± 82.8 days. Participants were censored (*n* = 29) if they did not participate in the CPS-3 2018 survey (*n* = 15), if they reported shiftwork in the 2018 survey (*n* = 12), or were pregnant (*n* = 2) in 2018.

**Figure A2:**
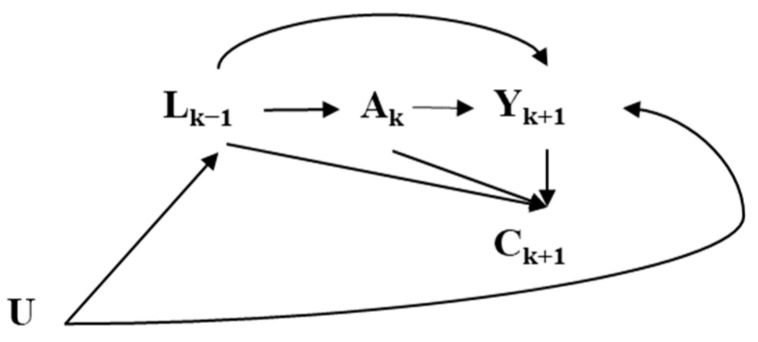
Simplified causal directed acyclic graph encoding the assumed relationships between the effect of treatment A on the outcome of interest Y and censoring C. Lk-1: covariates at time k − 1 (sex, race, income, education, work status, body mass index, diet quality, alcohol intake, fasting duration); Ak: exposure (treatment [fasting duration]) at time k; Yk + 1: outcome of interest (body weight) at time k + 1; Ck + 1: censoring at time k + 1; U: unmeasured risk factors for covariates L and outcome Y.

Third, to emulate random assignment to fasting strategies and ensure exchangeability, we used stabilized inverse probability weighing, which estimates the probability of fasting strategies given pre-baseline confounders such as age, sex, race, education, income, employment status, diet quality, alcohol consumption, sleep duration, BMI, and pre-baseline exposure. We used direct acyclic graphs to visually present the assumed causal relationship among those confounders, treatment, censoring, and body weight. (Figure A2) [32]. Under the assumption that the measured covariates are sufficient to adjust for both confounding and selection bias due to loss to follow-up, this simulates the conditions of a randomized controlled trial, where treatment assignment is independent of confounders [16,33].

### 2.4. Statistical Analyses

The observational analog of the per-protocol effect in the target trials was estimated using marginal structural models with stabilized inverse probability weights by creating a pseudo-population where the distribution of confounders is balanced across different treatment groups [16]. For each individual, a weight was calculated based on the inverse probability of receiving the treatment they actually received, adjusting for pre-baseline confounders (race, sex, education, age, work status, income, BMI, diet quality, alcohol intake, pre-baseline overnight fasting duration [for overnight treatment], before-sleep fasting duration [for before-sleep treatment], pre-baseline after-sleep fasting duration, and baseline sleep duration [for after-sleep treatment]). To address censoring, we estimated stabilized inverse probability weights for censoring using a method similar to that used for treatment weights. The censoring weights were calculated as the ratio of the probability of remaining uncensored given the treatment assignment to the probability of remaining uncensored given both treatment and covariates [16,34]. The product of stabilized treatment and censoring weights was truncated at the 99th percentile to reduce the influence of extreme values. Differences between the mean body weight under each fasting duration were estimated using the shortest fasting duration as the reference within each intervention group. Then, 95% confidence intervals (CIs) were calculated using nonparametric bootstrapping with 500 samples.

To understand how different subgroups respond to interventions, we performed stratified analyses by sex [35], BMI [36] (<25 kg/m^2^ and ≥25 kg/m^2^), and the midpoint of sleep [37] (before and after the sample median).

In our analysis, we excluded 51 participants with missing data (income [*n* = 5], education [*n* = 16], work status [*n* = 14], diet quality [*n* = 14]). To assess the impact of excluding participants with missing covariates, we (1) performed multiple imputations of missing covariate values and undertook the sensitivity analysis to evaluate the potential bias introduced by excluding participants with missing data [38]. (2) To mitigate the influence of outliers, we truncated treatment weights at the 99th percentile. We performed the analysis without truncation to understand how extreme weights affect the estimates. (3) We assessed whether sex modifies the relationship between diet quality, fasting duration, and body weight by introducing the interaction term between sex and diet quality in estimating treatment weights [39]. To explore the potential impact of physical activity on individual fasting duration habits [40], (4) we further adjusted our estimates for physical activity. We (5) further adjusted for the other fasting intervals in the before- and after-sleep fasting models. Finally, (6) we adjusted for the number of meals and snacks to account for missed eating occasions if participants had longer fasting durations.

All analyses were performed using SAS 9.4 software (SAS Institute, Inc., Cary, NC, USA).

The study protocol was approved by the institutional review boards of Emory University.

## 3. Results

### 3.1. Study Population

**Figure 1:**
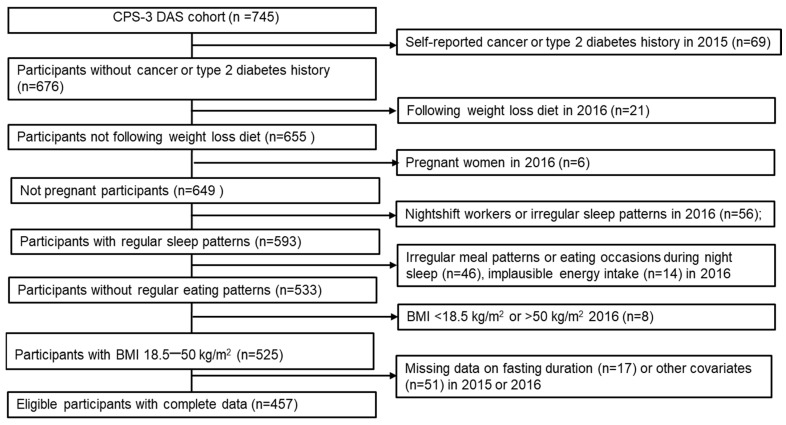
Flowchart of eligible individuals for the emulation of three target trial interventions in the CPS-3 DAS (2015–2018).

In the CPS-3 DAS cohort, 457 participants were eligible for the target trial emulation (Figure 1). The mean age of the participants was 51.2 years, with 35.4% being male, 22.2% Black, 14.5% Hispanic, and 63.3% White (Table 2). Most participants had a college degree or higher (79.2%), and the mean BMI was 27.1 kg/m^2^. At baseline, 57.5% reported <12 h of overnight fasting, 58.2% reported <4 h of before-sleep fasting, and 54.9% reported <1 h of after-sleep fasting. The mean baseline body weight was 78.3 kg.

### 3.2. Emulated Target Trial Estimates

After two years (median [IQR]: 2.0 [1.8–2.0] years), the estimated mean body weight under overnight fasting for the ≥12 h strategy was 79.4 kg, compared with 78.9 kg under <12 h (mean difference: 0.4 kg; 95% CI: −4.1 to 4.7) (Table 3). For before-sleep fasting, under the ≥4 h strategy, estimated weight was 79.4 kg vs. 77.5 kg under <4 h (mean difference: 1.9 kg; 95% CI: −0.4 to 4.1). For after-sleep fasting, under the ≥1 h strategy, the mean weight was 79.8 kg vs. 78.9 kg under the <1 h strategy (mean difference: 0.9 kg; 95% CI: −4.3 to 4.4).

### 3.3. Subgroup Analyses

Subgroup analyses stratified by sex, sleep midpoint, and BMI are summarized in Table 4. Among men, under the overnight ≥12 h strategy, weight was 100.9 kg vs. 83.9 kg under the <12 h strategy (mean difference: 17.0 kg; 95% CI: 10.8, 23.1). Among women, under the overnight ≥12 h strategy, weight was 74.3 kg vs. 77.1 kg under the <12 h strategy (mean difference: −2.8 kg; 95% CI: −6.8, 1.2). For participants with an earlier sleep midpoint (before 3:17 a.m.), overnight fasting (≥12 h) resulted in an estimated mean weight of 78.5 kg vs. 79.0 kg for <12 h (mean difference: −0.5 kg; 95% CI: −5.2, 4.2). In those with a later sleep midpoint (3:17 a.m. or later), mean weights were similar between fasting durations (80.8 kg for ≥12 h vs. 80.6 kg for <12 h; mean difference: 0.2 kg; 95% CI: −5.0, 5.5). Among participants with BMI <25 kg/m^2^, overnight fasting (≥12 h) resulted in an estimated mean weight of 66.5 kg vs. 65.7 kg for <12 h (mean difference: 0.8 kg; 95% CI: −2.3, 3.8). Among those with BMI ≥25 kg/m^2^, the estimated mean weight was 88.2 kg for ≥12 h vs. 89.8 kg for <12 h (mean difference: −1.6 kg; 95% CI: −6.0, 2.8). Similar small and non-significant differences were observed for before-sleep and after-sleep fasting strategies across all subgroups. Stratification by pre-baseline fasting exposure showed minimal differences in estimated mean body weight across fasting strategies (Table A1).

### 3.4. Sensitivity Analysis

The sensitivity analysis (Table A2) suggested that the estimates from the primary analysis (excluding participants with missing data) were consistent with those obtained from the imputed datasets, suggesting that excluding participants with the missing data had minimal effect on the estimates [41]. The truncation of the extreme weights did not impact the estimates. The mean inverse probability weights were 1.1 ± 0.1 before truncation and 1.0 + 0.1 after the truncation at the 99th percentile. Including an interaction term between sex and diet quality as well as further adjustment for physical activity, other fasting duration, and number of eating occasions had no major effect on the estimates.

## 4. Discussion

This study prospectively examines the effects of various fasting periods, particularly overnight fasting, before-sleep fasting, and after-sleep fasting, on body weight in a modern multiethnic CPS-3 DAS cohort. While we found little indication that the longer overnight fasting (≥12 h) strategy substantially influences the body weight in this population, we detected some differences by sex, suggesting that, compared to shorter overnight fasting (<12 h), longer overnight fasting may be linked to higher weight in men but has no effect in women. We did not detect differences in body weight linked to longer before- (≥4 h) or after-sleep (≥1 h) fasting.

Interestingly, among men assigned to the ≥12 h overnight fasting strategy, estimated body weight was higher than in those with shorter fasting. One plausible mechanism is hormone-mediated: fasting has been shown to lower circulating testosterone in men, which in turn can reduce lean mass and basal metabolic rate and thereby predispose to weight gain over time [42]. For example, earlier studies reported significant declines in testosterone following a time-restricted eating protocol [22], and low androgen levels were associated with adverse shifts in body composition [43,44]. Moreover, a trial of prolonged fasting in healthy young adults found that females, but not males, experienced lower body weight and improved body composition under extended fasting [45]. Together, these findings support that prolonged overnight fasting may differentially impact men’s metabolic health and body weight, and further investigation of the gender-related difference in the effect of fasting is needed.

Our results align with previous research that has shown mixed findings regarding the effectiveness of intermittent fasting for weight management [46,47,48]. For instance, a 12-week randomized weight-loss trial in individuals with overweight and obesity demonstrated no significant difference in weight loss among participants eating from 12:00 p.m. to 8:00 p.m. compared to a control group who was instructed to eat three meals per day at any time [46]. Another 12-week intervention on 19 participants with metabolic syndrome found that 10 h of time-restricted eating (14 h of overnight fasting) promoted weight loss and helped to reduce waist circumference and body fat [47]. Our null findings for extended before-sleep and after-sleep fasting mirror mixed evidence from studies of early dinner and delayed breakfast. A five-week crossover trial with 11 participants found that early time-restricted eating, where participants consumed all meals between 8:00 a.m. and 2:00 p.m., improved insulin sensitivity and appetite regulation more than a delayed eating schedule (eating between 12:00 p.m. and 8:00 p.m.) but did not necessarily lead to weight loss [49]. Another randomized trial of TRE found that early TRE (8-h eating window between 7:00 a.m. and 3:00 p.m.) may be more effective for weight loss but not for improving body fat composition or other metabolic factors, compared to eating anytime during a self-selected ≥12-h window [50]. Together, these findings suggest that isolated shifts in the timing of the last or first meal may not substantially influence body weight without changes in total energy intake, meal composition, or circadian factors.

One of the strengths of our study is the use of an emulated target trial design with explicitly defined treatment strategies and stabilized inverse probability weighting, allowing for a rigorous comparison of different fasting strategies using observational data [51]. The large, diverse cohort from the CPS-3 DAS provides a robust sample larger than most randomized trials. We also assessed the effect of intervention in participants who did not self-report prolonged fasting before baseline for at least 12 months, allowing for reducing the prevalent user bias and ensuring a more accurate comparison between different fasting strategies [52].

There are several limitations to acknowledge. First, self-reported data on fasting durations and body weight may be subject to recall bias and measurement error. The self-reported body weight was validated against in-person measurements from previous studies, ensuring reliability [26]. Similarly, meal timing and sleep grid were shown to be reliable tools for the assessment of fasting duration [21]. Second, our study design assumes that fasting habits remained consistent over the follow-up, which may not accurately reflect participants’ actual behavior and may not allow us to distinguish between per-protocol and intention-to-treat effects. While more frequent, repeated data collection could enhance this study’s strengths; the reported data from the meal timing grid was confirmed to have a relatively high one-year reproducibility with ρ = 0.62, suggesting that it is reasonable to believe that most of the participants sustained their reported fasting durations for 12 months before the end of follow-up [21]. While we excluded the participants with diabetes and cancer from the analytic cohort, our analysis did not account for the change in diabetes status or other comorbidities during the follow-up. Although we adjusted for a comprehensive list of confounders, the emulated target trial relies on several assumptions and is unable to eliminate uncontrolled confounding of the variables not assessed in the CPS-3 cohort. To assess the effect of the analytic decision made in our study, we conducted an extensive sensitivity analysis, which produced results similar to those of our primary analysis. Although we have adjusted each IPW model for the other fasting intervals, and we found no correlation between baseline before and after-sleep fasting durations (Spearman ρ = –0.06), our ability to disentangle the independent effects of before- and after-sleep fasting durations may still be limited. Finally, although social jetlag [53] (the mismatch between sleep timing on workdays and weekends) may influence both fasting timing and body weight, our meal-and-sleep grid lacked the day-to-day resolution to quantify a valid social-jetlag metric, and our sample size is likely insufficient to robustly assess its confounding effect in IPW models.

## 5. Conclusions

In conclusion, our findings suggest that longer overnight fasting alone may not substantially influence body weight, but sex-specific differences need to be further investigated. Larger future studies and stronger methods to longitudinally document behavior in real time are needed to investigate the joint effect of overnight fasting and other dietary and behavioral factors, like physical activity and energy intake, on body weight.

## Figures and Tables

**Table 1:** Emulation of the target trials of overnight fasting interventions using observational data from the CPS-3 DAS.

Component	Target Trial Specifications	Target Trial Emulation
Aim	Estimate the effect of overnight fasting, before-sleep fasting, and after-sleep fasting strategies on body weight in U.S. adults across three independent interventions.	Same as target trial.
Eligibility criteria	Exclude: Night shift workers. Irregular sleep patterns (night sleep < 4 or >14 h, starting night sleep after 5:00 a.m. or before 5:00 p.m.). Consuming meals or snacks during night sleep. BMI outside < 18.5 or >50 kg/m^2^. Currently pregnant. Cancer or diabetes history. On a weight-loss diet. Implausible energy intake. Irregular eating pattern (≤1 or >8 meals/day).	Same as target trial. Additional exclusion criteria: Missing data on fasting duration or other covariates at pre-baseline or baseline.
Treatment strategies	Individuals would be assigned to one of the following strategies: <12 h of overnight fasting. ≥12 h of overnight fasting. <4 h of fasting before sleep. ≥4 h of fasting before sleep. <1 h of fasting after sleep. ≥1 h of fasting after sleep.	Same as target trial.
Treatment assignment	Random assignment at baseline (2016), with strategy awareness. Treatment starts at assignment and lasts 1 year with monthly adherence checks.	Emulate randomization by adjusting for pre-baseline confounders (race, sex, education, age, work status, income BMI, diet quality, alcohol intake, and respective pre-baseline fasting durations). No adherence assessment during the follow-up.
Follow-up	Follow-up begins from baseline (2016) when an individual is assigned to treatment. Follow-up ends at the earliest of death, loss to follow-up, or administrative end of follow-up 2 years since baseline. Participants are censored if they did not participate in the follow-up, reported pregnancy, or shift work.	Follow-up begins from baseline when an individual returns the CPS-3 DAS 2016 survey. Follow-up ends at the earliest of death, loss to follow-up, or the administrative end of follow-up (the return date of the self-administered CPS-3 2018 survey). Participants are censored if they did not participate in the CPS-3 outcome assessment survey, reported pregnancy, or shift work in 2018.
Outcome	Body weight (kg) assessed by self-administered questionnaire in 2018.	Same as target trial.
Causal contrast of interest	Intention-to-treat effect. Per-protocol effect.	Cannot distinguish between observational analog of intention-to-treat effect and observational analog of per-protocol effect.
Statistical analyses	Intention-to-treat analysis. Per-protocol analysis: Marginal structural models with stabilized inverse probability weight to compare body weight between treatment groups in each intervention, adjusting for baseline and pre-baseline factors related to adherence and censoring. Non-parametric bootstrap for 95% CIs.	Same as the per-protocol analysis in the target trial.

**Table 2:** Baseline characteristics of eligible participants for overnight, before-sleep, and after-sleep fasting emulated target trials using the CPS-3 DAS data.

		Total	Overnight Fasting		Before-Sleep Fasting		After-Sleep Fasting	
Characteristics			<12 h	≥12 h	<4 h	≥4 h	<1 h	≥1 h
		*n* = 457	*n* = 263	*n* = 194	*n* = 266	*n* = 191	*n* = 251	*n* = 206
Fasting duration range			8.1–11.9 h	12.1–17.6 h	1.1–3.9 h	4.1–9.5 h	0–0.9 h	1.0–8.1 h
Age, y		51.2 (9.63)	51.4 (9.68)	51.1 (9.59)	51.2 (9.45)	51.4 (9.9)	51.7 (9.93)	50.7 (9.24)
Male, *n* (%)		162 (35.4)	103 (39.2)	59 (30.4)	87 (32.7)	75 (39.3)	94 (37.5)	68 (33)
Race/ethnicity, *n* (%)	Black	103 (22.5)	57 (21.7)	46 (23.7)	57 (21.4)	46 (24.1)	38 (15.1)	65 (31.6)
	Hispanic	63 (13.8)	38 (14.4)	25 (12.9)	35 (13.2)	28 (14.7)	37 (14.7)	26 (12.6)
	White	291 (63.7)	168 (63.9)	123 (63.4)	174 (65.4)	117 (61.3)	176 (70.1)	115 (55.8)
Education, *n* (%)	High school/some college	95 (20.8)	47 (17.9)	48 (24.7)	56 (21.1)	39 (20.4)	46 (18.3)	49 (23.8)
	College degree	166 (36.3)	95 (36.1)	71 (36.6)	103 (38.7)	63 (33)	92 (36.7)	74 (35.9)
	Graduate degree	196 (42.9)	121 (46)	75 (38.7)	107 (40.2)	89 (46.6)	113 (45)	83 (40.3)
Income, *n* (%)	<25 K	16 (3.5)	8 (3)	8 (4.1)	6 (2.3)	10 (5.2)	8 (3.2)	8 (3.9)
	25–<50 K	55 (12)	24 (9.1)	31 (16)	29 (10.9)	26 (13.6)	20 (8)	35 (17)
	50–<75 K	67 (14.7)	44 (16.7)	23 (11.9)	43 (16.2)	24 (12.6)	38 (15.1)	29 (14.1)
	75–<100 K	62 (13.6)	31 (11.8)	31 (16)	33 (12.4)	29 (15.2)	33 (13.1)	29 (14.1)
	≥100 K	257 (56.2)	156 (59.3)	101 (52.1)	155 (58.3)	102 (53.4)	152 (60.6)	105 (51)
Married or living with partner, *n* (%)		335 (73.3)	199 (75.7)	136 (70.1)	201 (75.6)	134 (70.2)	193 (76.9)	142 (68.9)
Current smoker, *n* (%)		14 (3.1)	7 (2.7)	7 (3.6)	5 (1.9)	9 (4.7)	8 (3.2)	6 (2.9)
Employed, *n* (%)		375 (82.1)	227 (86.3)	148 (76.3)	226 (85)	149 (78)	210 (83.7)	165 (80.1)
Post-menopausal females ^2^, *n* (%)		116 (39.3)	64 (40.0)	52 (38.5)	73 (40.8)	43 (37.1)	60 (38.2)	56 (40.6)
BMI, kg/m^2^		27.1 (5.55)	26.7 (5.5)	27.6 (5.57)	26.9 (5.38)	27.4 (5.78)	26.3 (5.11)	28.1 (5.9)
HEI-2015 score ^1^		71.5 (7.84)	71.9 (7.96)	71 (7.67)	71.2 (7.52)	71.8 (8.28)	72.8 (7.36)	70 (8.14)
Alcohol intake, mL/wk		9.2 (13.71)	8.8 (12.73)	9.8 (14.94)	8.7 (12.66)	10 (15.03)	8.6 (12.53)	10 (15.01)
Energy intake, kcal/d		2023.9 (638.1)	2065.2 (663.9)	1967.9 (598.5)	2022.6 (611.0)	2025.7 (675.6)	2049.6 (665.5)	1992.5 (603.2)
Physical activity, MET-h/wk		6.6 (5.88)	6.7 (5.93)	6.4 (5.81)	6.4 (5.68)	6.9 (6.14)	7.2 (5.78)	5.8 (5.91)
Sleep duration, h		7.9 (1.02)	7.7 (0.98)	8.1 (1.01)	8 (0.96)	7.7 (1.07)	8.1 (0.97)	7.7 (1.04)
Sleep midpoint ^3^, HH:MM		03:18	03:18	03:24	03:12	03:36	03:24	03:18
Snacks, snacks/day		1.73 (0.89)	1.94 (0.90)	1.45 (0.81)	1.99 (0.85)	1.37 (0.83)	1.73 (0.92)	1.74 (0.86)
Meals, meals/day		2.80 (0.42)	2.88 (0.38)	2.70 (0.45)	2.84 (0.42)	2.74 (0.42)	2.86 (0.37)	2.73 (0.46)
Overnight fasting, *n* (%)	<12 h	263 (57.5)			200 (75.2)	63 (33)	181 (72.1)	82 (39.8)
	≥12 h	194 (42.5)			66 (24.8)	128 (67)	70 (27.9)	124 (60.2)
Before-sleep fasting, *n* (%)	<4 h	266 (58.2)	200 (76)	66 (34)			139 (55.4)	127 (61.7)
	≥4 h	191 (41.8)	63 (24)	128 (66)			112 (44.6)	79 (38.3)
After-sleep fasting, *n* (%)	<1 h	251 (54.9)	181 (68.8)	70 (36.1)	139 (52.3)	112 (58.6)		
	≥1 h	206 (45.1)	82 (31.2)	124 (63.9)	127 (47.7)	79 (41.4)		
Body weight, kg		78.3 (17.9)	77.8 (17.7)	79.1 (18.2)	77.5 (17.3)	79.6 (18.7)	76.4 (17.3)	80.7 (18.4)

Values are mean (SD) or *n* (%). ^1^ Total HEI-2015 score is out of 100. ^2^ Percentage is out of 307 females. ^3^ Sleep midpoint is the time halfway between the start and the end of the night sleep (longest sleep period).

**Table 3:** Estimated mean body weight (kg) under overnight, before-sleep, and after-sleep fasting strategies in the emulated target trials in the CPS-3 DAS (2015–2018).

Intervention Strategy	Mean Body Weight ^1,2^ (kg) (95% CI)	Mean Difference (kg) (95% CI)
Overnight fasting		
<12 h	78.9 (75.5, 82.9)	0 (Referent)
≥12 h	79.4 (76.2, 82.2)	0.4 (−4.1, 4.7)
Before-sleep fasting		
<4 h	77.5 (75.5, 79.3)	0 (Referent)
≥4 h	79.4 (76.9, 81.5)	1.9 (−0.4, 4.1)
After-sleep fasting		
<1 h	78.9 (75.6, 84.1)	0 (Referent)
≥1 h	79.8 (77.7, 81.9)	0.9 (−4.3, 4.4)

^1^ Marginal structural models with stabilized inverse probability weighting adjusted for race, sex, education, age, work status, income, pre-baseline BMI, diet quality, alcohol intake, and respective pre-baseline fasting durations. Bootstrapping with 500 samples was used to obtain 95% confidence intervals. ^2^ Observed mean body weight was 78.6 ± 18.0 kg.

**Table 4:** Estimated mean body weight (kg) under hypothetical fasting treatment strategies in subgroup analyses in the CPS-3 DAS (2015–2018).

Intervention Strategy	Estimated Mean ^1^ and Mean Difference (95% CI) (kg), Stratified by Baseline Covariates			
**Stratified by Sex ^3^**	**Male (*n* = 162)**		**Female (*n* = 295)**	
<12 h overnight fasting	83.9 (80.4, 87.5)	0 (Referent)	77.1 (74.4, 79.7)	0 (Referent)
≥12 h overnight fasting	100.9 (95.9, 105.9)	17.0 (10.8, 23.1)	74.3 (71.3, 77.3)	−2.8 (−6.8, 1.2)
<4 h before-sleep fasting	84.6 (81.1, 88.1)	0 (Referent)	73.6 (71.1, 76.1)	0 (Referent)
≥4 h before-sleep fasting	87.1 (83.3, 90.9)	2.5 (−2.6, 7.6)	75.4 (72.3, 78.5)	1.9 (−2.1, 5.8)
<1 h after-sleep fasting	83.4 (80.4, 86.5)	0 (Referent)	77.6 (74.6, 80.6)	0 (Referent)
≥1 h after-sleep fasting	87.8 (84.1, 91.5)	4.4 (−0.4, 9.2)	75.2 (71.7, 78.8)	−2.4 (−7.0, 2.3)
**Stratified by sleep midpoint ^2,4^**	**Before 3:17 a.m. (*n* = 235)**		**3:17 a.m. or later (*n* = 222)**	
<12 h overnight fasting	79.0 (76.1, 81.9)	0 (Referent)	80.6 (77.1, 84.1)	0 (Referent)
≥12 h overnight fasting	78.5 (74.7, 82.2)	−0.5 (−5.2, 4.2)	80.8 (76.9, 84.7)	0.2 (−5.0, 5.5)
<4 h before-sleep fasting	75.5 (72.7, 78.3)	0 (Referent)	80.1 (76.6, 83.5)	0 (Referent)
≥4 h before-sleep fasting	79.7 (75.8, 83.7)	4.2 (−0.6, 9.1)	78.8 (75.5, 82.2)	−1.2 (−6.0, 3.5)
<1 h after-sleep fasting	79.5 (76.1, 82.8)	0 (Referent)	80.3 (77.0, 83.6)	0 (Referent)
≥1 h after-sleep fasting	78.2 (74.6, 81.9)	−1.2 (−6.2, 3.7)	82.5 (78.4, 86.6)	2.2 (−3.1, 7.5)
**Stratified by BMI ^5^**	**<25 kg/m^2^ (*n* = 204)**		**≥25 kg/m^2^ (*n* = 253)**	
<12 h overnight fasting	65.7 (64.0, 67.5)	0 (Referent)	89.8 (86.9, 92.7)	0 (Referent)
≥12 h overnight fasting	66.5 (64.0, 68.9)	0.8 (−2.3, 3.8)	88.2 (84.9, 91.5)	−1.6 (−6.0, 2.8)
<4 h before-sleep fasting	66.1 (64.4, 67.8)	0 (Referent)	87.4 (84.6, 90.2)	0 (Referent)
≥4 h before-sleep fasting	67.2 (65.1, 69.3)	1.1 (−1.6, 3.8)	89.3 (86.3, 92.4)	1.9 (−2.3, 6.0)
<1 h after-sleep fasting	66.5 (64.8, 68.2)	0 (Referent)	89.1 (85.9, 92.3)	0 (Referent)
≥1 h after-sleep fasting	68.5 (66.3, 70.8)	2.1 (−0.8, 4.9)	88.2 (85.1, 91.4)	−0.8 (−5.3, 3.7)

^1^ Marginal structural models with stabilized inverse probability weighting adjusted for race, sex, education, age, work status, income, pre-baseline BMI, diet quality, alcohol intake, and respective pre-baseline fasting durations. Bootstrapping with 500 samples was used to obtain 95% confidence intervals. ^2^ Sleep midpoint is the time halfway between the start and end of night sleep. In the sample, 3:17 a.m. is a median sleep midpoint. ^3^ Observed mean body weight was 86.4 ± 16.6 kg among males and 74.1 ± 17.0 kg among females. ^4^ Observed mean body weight was 77.4 ± 17.8 kg among individuals who had sleep midpoint before 3:17 a.m. and 80.5 ± 18.3 kg among individuals who had sleep midpoint at 3:17 a.m. or later. ^5^ Observed mean body weight was 66.4 ± 9.7 kg among individuals with BMI < 25 kg/m^2^ and 88.8 ± 16.9 kg among individuals with BMI ≥ 25 kg/m^2^.

## Data Availability

Data are available from the American Cancer Society by following the ACS Data Access Procedures (“https://www.cancer.org/content/dam/cancer-org/research/epidemiology/cancer-prevention-study-data-access-policies.pdf” [accessed on 29 April 2025]) for researchers who meet the criteria for access to confidential data. Please email cohort.data@cancer.org to inquire about access.

## Abbreviations

The following abbreviations are used in this manuscript:

CPS-3 DAS	Cancer Prevention Study-3 The Diet Assessment Substudy
MET	Metabolic Equivalent of Task
BMI	Body Mass Index

## Appendix A

**Table A1:** Estimated mean body weight (kg) under hypothetical fasting treatment strategies by pre-baseline exposure in the CPS-3 DAS (2015–2018).

Intervention Strategy	Estimated Mean ^1^ and Mean Difference (95% CI) (kg), Stratified by Pre-Baseline Fasting Exposure			
**Stratified by overnight fasting ^2^**	**<12 h (*n* = 246)**		**≥12 h (*n* = 211)**	
<12 h overnight fasting	79.4 (76.9, 82.2)	0 (Referent)	77.8 (73.5, 84.1)	0 (Referent)
≥12 h overnight fasting	82.5 (74.2, 93.6)	3.1 (−5.0, 14.3)	78.3 (75.6, 81.0)	0.6 (−6.7, 5.2)
**Stratified by before-sleep fasting ^3^**	**<4 h (*n* = 230)**		**≥4 h (*n* = 227)**	
<4 h before-sleep fasting	78.6 (76.4, 81.0)	0 (Referent)	76.3 (73.3, 79.5)	0 (Referent)
≥4 h before-sleep fasting	79.9 (76.4, 83.4)	1.4 (−2.3, 5.1)	79.2 (76.0, 82.0)	2.9 (−0.4, 5.8)
**Stratified by after-sleep fasting ^4^**	**<1 h (*n* = 239)**		**≥1 h (*n* = 218)**	
<1 h after-sleep fasting	78.4 (75.7, 82.6)	0 (Referent)	77.4 (73.4, 81.4)	0 (Referent)
≥1 h after-sleep fasting	77.5 (73.9, 80.9)	−0.9 (−5.3, 3.0)	80.8 (77.6, 83.8)	3.4 (−1.6, 7.5)

^1^ Marginal structural models with stabilized inverse probability weighting adjusted for race, sex, education, age, work status, income, pre-baseline BMI, diet quality, alcohol intake, and respective pre-baseline fasting durations. Bootstrapping with 500 samples was used to obtain 95% confidence intervals. ^2^ Observed mean body weight was 79.2 ± 17.6 kg among individuals who reported <12 h overnight fasting and 77.9 ± 18.5 kg among individuals who reported ≥12 h overnight fasting at pre-baseline. ^3^ Observed mean body weight was 78.8 ± 17.4 kg among individuals who reported <4 h before-sleep fasting and 78.5 ± 18.5 kg among individuals who reported ≥4 h before-sleep fasting at pre-baseline. ^4^ Observed mean body weight was 77.6 ± 17.2 kg among individuals who reported <1 h after-sleep fasting and 80.0 ± 18.9 kg among individuals who reported ≥1 h after-sleep fasting at pre-baseline. Abbreviations: BMI, Body Mass Index; CPS-3 DAS, Cancer Prevention Study-3 The Diet Assessment Substudy.

**Table A2:** Sensitivity analysis. Estimated mean body weight (kg) under overnight, before-sleep, and after-sleep fasting strategies in the emulated target trials in the CPS-3 DAS (2015–2018).

Sensitivity Analysis	Estimated Mean Body Weight ^1^ (95% CI)/Mean Difference (95% CI) (kg)					
	Overnight Fasting		Before-Sleep Fasting		After-Sleep Fasting	
	<12 h	≥12 h	<4 h	≥4 h	<1 h	≥1 h
0. Primary	78.9 (75.5, 82.9)/Reference	79.4 (76.2, 822)/0.4 (−4.1, 4.7)	77.5 (75.7, 79.3)/Reference	79.4 (76.9, 81.5)/1.9 (−0.4, 4.1)	78.9 (75.6, 84.1)/Reference	79.8 (77.6, 81.9)/0.9 (−4.3, 4.4)
1. Imputation ^2,3^	80.5 (77.5, 83.4)/Reference	79.7 (76.6, 83.2)/−0.8 (−4.0, 3.0)	78.1 (76.4, 80.0)/Reference	80.0 (78.0, 82.2)/1.9 (−0.1, 3.9)	79.6 (76.6, 83.3)/Reference	79.5 (77.4, 81.7)/−0.1 (−3.6, 3.1)
2. Truncation ^4^	79.7 (75.4, 83.9)/Reference	79.6 (76.4, 82.2)/−0.1 (−4.6, 4.6)	77.6 (75.7, 79.2)/Reference	79.4 (76.9, 81.5)/1.8 (−0.4, 4.0)	78.6 (72.6, 89.3)/Reference	79.8 (77.6, 81.9)/1.2 (−9.2, 7.1)
3. Interaction ^5^	79.1 (75.6, 82.7)/Reference	79.3 (75.9, 82.4)/0.5 (−3.9, 4.5)	77.5 (75.6, 79.3)/Reference	79.4 (76.9, 81.6)/1.9 (−0.4, 4.1)	78.9 (75.7, 82.0)/Reference	80.0 (77.9, 81.9)/0.8 (−4.6, 4.4)
4. Further adjustment for physical activity ^6^	78.7 (73.5, 86.0)/Reference	79.2 (74.3, 83.6)/0.4 (−8.7, 7.3)	77.0 (74.7, 79.0)/Reference	80.3 (76.9, 84.4)/3.3 (−0.1, 8.1)	78.5 (74.3, 85.6)/Reference	79.5 (75.9, 83.2)/1.0 (−7.1, 6.4)
5. Further adjustment for before- or after-sleep durations ^7^	-	-	77.5 (75.5, 79.4)/Reference	79.4 (76.9, 81.5)/1.9 (−0.4, 4.1)	78.9 (75.7, 84.0)/Reference	79.8 (77.7, 81.9)/0.9 (−4.0, 4.5)
6. Further adjustment for the number of eating occasions ^8^	79.0 (75.4, 83.3)/ Reference	79.3 (76.0, 82.3)/0.3 (−4.7, 4.7)	77.4 (75.5, 79.2)/ Reference	79.4 (77.0, 81.6)/2.0 (−0.4, 4.2)	78.9 (75.7, 84.6)/ Reference	79.9 (77.8, 82.0)/1.0 (−4.2, 4.5)

^1^ Marginal structural models with stabilized inverse probability weighting adjusted for race, sex, education, age, work status, income, pre-baseline BMI, diet quality, alcohol intake, and respective pre-baseline fasting durations. Bootstrapping with 500 samples was used to obtain 95% confidence intervals. ^2^ Observed mean body weight was 79.3 ± 18.4 kg. ^3^ Multiple imputations with 20 samples were used to impute missing income, education, work status, and diet quality values assessed at pre-baseline. ^4^ Stabilized inverse probability weights were not truncated. ^5^ Interaction term between sex and diet quality was included in the models to estimate stabilized weights. ^6^ Physical activity was measured as moderate-to-vigorous activity volume at pre-baseline. ^7^ In the before-sleep fasting intervention, we further adjusted for pre-baseline after-sleep fasting durations. Similarly, we adjusted for before-sleep fasting in the after-sleep fasting intervention. ^8^ Number of eating occasions is a weighted average of the number of meals and snacks per week assessed at pre-baseline.
